# Clinical teaching practices of nurse educators: An integrative literature review

**DOI:** 10.4102/hsag.v27i0.1728

**Published:** 2022-09-30

**Authors:** Sybil N. Gcawu, Dalena van Rooyen

**Affiliations:** 1 Department of Nursing Science, Faculty of Health Sciences, Nelson Mandela University, Port Elizabeth, South Africa; 2 Faculty of Health Sciences, Nelson Mandela University, Port Elizabeth, South Africa

**Keywords:** clinical teaching practice, nurse educator, clinical educator, clinical teacher, preceptor, mentor, undergraduate nursing student, variation

## Abstract

Clinical teaching practice of nurse educators is important in the development of clinical competence of undergraduate nursing students, but it is often not done according to best practice standards. This study aimed to summarise the best clinical teaching practices of nurse educators teaching undergraduate nursing programmes. An integrative literature review was conducted according to Whittemore and Knafl’s adapted stages. A systematic search of electronic databases, including EBSCOhost and ScienceDirect, for applicable papers from January 2001 to June 2021, was followed by a manual search. The review resulted in selection of 67 papers, and critical appraisal had been completed by two independent reviewers using relevant critical appraisal tools. Papers that were selected revealed six main themes, with sub-themes, outlining best practices for clinical teaching, namely: (1) planning for clinical teaching practice: self-preparation and planning for clinical placement; (2) facilitation of students’ clinical learning: orientation of students, planning for clinical teaching and the clinical teaching process; (3) evaluation of students’ clinical skills: reflection by the nursing student and feedback; (4) modelling professional clinical teaching practice: emotional intelligence, self-evaluation, role modelling and continuous professional development; (5) work-based assessment in the clinical environment: clinical assessment process and clinical assessment tools and (6) clinical teaching in the simulation laboratory. The six identified best practices could assist nurse educators to provide comprehensive clinical teaching.

**Contribution:**

The review’s results can be used in the development of a best practice guideline for clinical teaching. This guideline will aid nurse educators in achieving best clinical teaching practices.

## Introduction

Clinical education of undergraduate nurses remains an integral part of the nursing curriculum and forms the foundation for bridging the theory-practice gap (Wells & McLoughlin 2014). Therefore, the nursing curriculum needs to be aligned to the clinical setting to ensure that graduates are equipped to face the challenges of complex and dynamic healthcare delivery system (Bvumbwe 2016). Literature suggests that the process of clinical teaching begins with identification of the goals and outcomes for clinical learning, and proceeds through planning clinical learning activities, guiding students, assessing the learner and evaluating clinical learning and performance (Gaberson, Oermann & Shellenbarger 2017).

The clinical teaching role of the nurse educator encompasses guidance, support, stimulation and facilitation of learning in the range of practice settings, which include hospitals, clinics and other primary healthcare sites (World Health Organisation 2016). In the process, undergraduate nursing students get the opportunity to practise nursing care, acquire the necessary competencies, internalise professional values and develop their interpersonal skills (Gaberson et al. 2017).

One of the responsibilities of the nurse educator is to convey theoretical knowledge to the nursing students in clinical practice, thus ensuring integration of theory and practice. Although some reviews have been conducted regarding teaching strategies or clinical teaching practices of nurse educators, these reviews focussed on classroom teaching of theory (Breytenbach, Ten Ham-Baloyi & Jordan 2017) or the use of research by nurse educators in clinical teaching (Milner, Estabrooks & Myrick 2006). Summarising literature regarding clinical teaching practices of nurse educators would assist (novice) nurse educators to identify relevant clinical teaching practices and to set a foundation for standardisation and professionalism in clinical teaching practices to achieve the best possible learning outcomes for undergraduate nursing students (Gaberson et al. 2017). Furthermore, according to the authors’ knowledge, no integrative literature review has been published, summarising the clinical teaching practices of nurse educators, teaching undergraduate nursing programmes, indicating a need for such a review to be conducted.

## Methods

The integrative literature review was conducted in five stages, adapted from Whittemore and Knafl (2005). These were: Stage 1: Problem identification; Stage 2: Literature search; Stage 3: Data evaluation; Stage 4: Data analysis and Stage 5: Presentation.

The review question derived from the identified problem was formulated as follows: *What are the clinical teaching practices of nurse educators teaching undergraduate nursing programmes?*

### Literature search

The literature search was conducted by the first author under the supervision of the second author. The university librarian assisted with the search strategy, including the identification of sources of literature and key words, as described in the following subsection.

### Sources of literature

The EBSCOhost search engine was used to search for literature from the following databases: Cumulative Index for Nursing and Allied Health Literature (CINAHL); Education Resources Information Centre (ERIC); Medical Literature Analysis & Retrieval System Online (MEDLINE); E-Journals; Health Sources: Nursing/Academic edition; Master File Premier; Teacher Reference Centre as well as ScienceDirect. Subsequently, a manual search of grey literature (unpublished papers) using Google Scholar and Google Search engines, as well as citation searching through reference lists, was conducted.

### Key words

The following key words were used to search for literature from databases: clinical teaching practice* AND clinical educat* OR nurse educ* OR clinical teach* OR mentor* OR preceptor* AND undergraduate*.

### Inclusion and exclusion criteria

Both research papers and non-research papers, such as opinion papers, and clinical practice guidelines reporting on the clinical teaching practices of nurse educators, including clinical teachers, mentors, preceptors and clinical educators, teaching undergraduate nursing programmes were included. Inclusion was limited to papers in English as this was the language the researcher was proficient in and dated between January 2001 and June 2021 to obtain sufficient evidence.

Papers that were included related to aspects of clinical teaching, namely: planning for clinical teaching practice, facilitation of nursing students’ clinical learning, evaluation of students’ clinical skills assessment, modelling professional clinical teaching, work-based assessment in the clinical environment and clinical teaching in the simulation laboratory.

The following papers were excluded: papers which were not relevant to the research study, such as those pertaining to clinical teaching practices by non-nursing educators (e.g. medical, dental and other non-nursing educators), teaching of theory, clinical teaching in postgraduate programmes and other health programmes, duplicated papers, papers written in other languages and those possibly relevant papers that could not be obtained.

### Documenting the search and selection process

**FIGURE 1:**
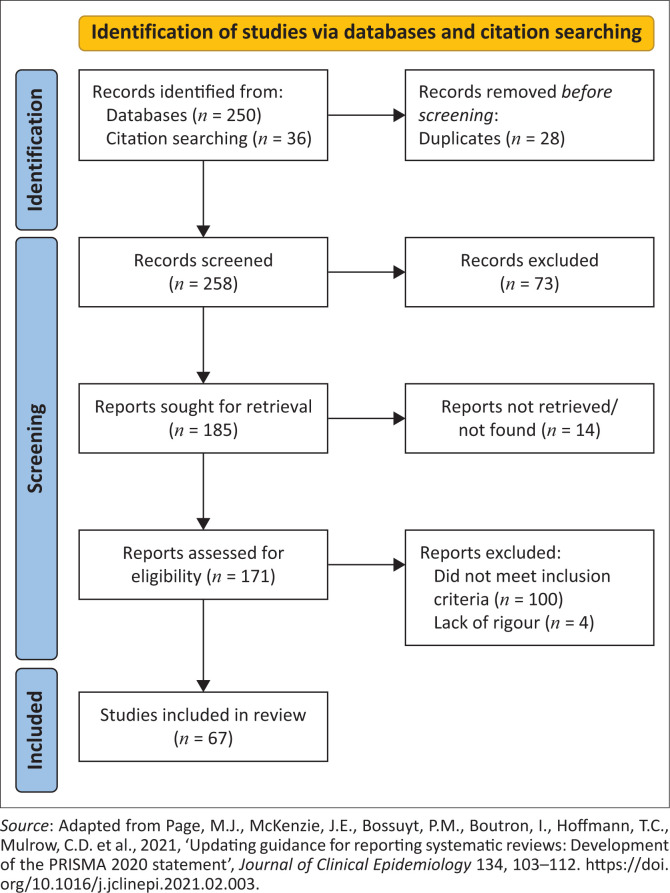
PRISMA: Search and selection process.

After titles and abstracts were read and selected for inclusion, based on the inclusion and exclusion criteria by both authors independently, full-texts were obtained for possible relevant literature. Full-texts were read and selected based on the inclusion and exclusion criteria. The search and selection process is reflected in Figure 1.

As reflected in Figure 1, the search through electronic databases yielded 286 papers – 250 through the databases and 36 papers through the manual search using citation searching. After manually removing 28 duplicate papers which had the same titles and/or abstracts, 258 records were scrutinised for relevance by titles and abstracts. Seventy-three records were excluded and 185 full-texts were sought for retrieval. Although interlibrary loan was used to retrieve as many full-texts as possible, a total of 14 papers could not be obtained as these papers were not accessible without payment because of non-subscription of the universities to the respective journals they were published in, resulting in 171 full-texts being read. A total of 100 papers did not meet the inclusion criteria and, after critical appraisal, four papers were excluded, leaving 67 papers to be included for data extraction and synthesis.

### Data evaluation

Various critical appraisal tools were used to assess the rigour of the papers extracted in view of the heterogeneity thereof. These tools included Johns Hopkins’ tools for mixed methods and literature reviews; the Joanna Briggs Institute for interpretive studies; the Assessment of Multiple Systematic Reviews (AMSTAR) tool and Critical Appraisal Skills Programme (CASP) for integrative review, qualitative and quantitative studies. The non-research papers were assessed using the Joanna Briggs Institute for the Narrative, Opinion and Text Assessment critical appraisal tool (NOTARI), and the guideline was assessed using the Rapid Best Practice Guideline appraisal tool from Melnyk and Fineout-Overholt (2011:518).

The methodological quality of all the papers extracted was assessed using the US Preventive Services Task Force (USPSTF 2012) Guide to clinical preventive services grade rating. This guide was selected in view of its ability to incorporate all levels of evidence from heterogeneous papers into a usable grade rating.

In order to enhance rigour of the integrative literature review process, the critical appraisal of data was performed by the first author and an independent reviewer. Research papers were included if they were considered to be of good or medium rigour according to the various quality appraisal instruments. Research papers generally had to comply with more than half of the following criteria: the aim is clearly stated, the research design is appropriate; sampling, data collection and data analysis are clearly described; findings, recommendations and limitations were clearly stated and ethical issues were considered. Similarly, non-research papers were included if they complied with more than half of the following criteria: whether the opinion of the author(s) was based on scientific evidence or evidence was based on the opinions of more than one individual, who are preferably experts on the topic; the conclusions and recommendations are provided, including potential biases and whether results can be applied and are relevant to clinical practice. The two reviewers discussed this process, and, when consensus was reached, the final selection of papers was made. Four of the 71 papers that were appraised were excluded owing to weak methodological rigour. A total of 67 papers were included for data extraction.

### Data analysis

Data analysis included the data extraction and synthesis. Data (including the reference, aim, method as well as findings relevant to the topic of each paper) were extracted by the first author from the 67 papers using the data extraction tool adapted from Russell (2005), in alignment with the review question. Extracted data were synthesised, using thematic analysis. Thematic results are presented in narrative form in the following section.

### Ethical considerations

This review was part of a doctoral study (ethics number: H15-HEA-NUR-008) that formulated the best practice guideline for clinical teaching at a public college of nursing. Consent to conduct the research was not obtained as this study had human participants.

## Findings

Sixty-seven (*n* = 67) papers were included. Of these, one was Randomised Control Trial, thirteen (13) were non-experimental interpretive studies, five (5) were literature reviews, thirty-two (32) were single descriptive or qualitative studies and fifteen (15) were non-research opinion papers of experts and reports of expert committees.

Papers reported research conducted in Canada (*n* = 11), the United States of America (*n* = 10), Australia (*n* = 9), the United Kingdom (*n* = 8), Ireland (*n* = 7), Iran (*n* = 5), Sweden (*n* = 4), Brazil (*n* = 2) and South Africa (*n* = 2). One paper was included from each of the following countries: Belgium, Finland, Israel, Japan, Malawi, Pakistan, Palestine, Scotland and Turkey. Based on the thematic analysis of the extracted data, six themes emerged from the data, namely: planning for clinical teaching practice; facilitation of students’ clinical placements; evaluation of students’ clinical skills; modelling professional clinical teaching; work-based assessment in the clinical environment and clinical teaching in the simulation laboratory. The results of thematic analysis are discussed in the following subsection.

### Theme One: Planning for clinical teaching practice

Adequate planning for clinical teaching, as mentioned by four papers (Lichtman et al. 2003; Luhanga 2018; Parkinson 2016; RNAO 2016) should cover aspects of self-preparation by the nurse educator and planning for placement of nursing students to the clinical sites.

The expectation in terms of *self-preparation* is for the nurse educator to be competent in terms of clinical teaching, and knowledgeable in terms of the clinical environment, prior to conducting clinical teaching (Parkinson 2016:9). In order to gain the required competencies, nurse educators should receive adequate preparation and support through professional development (Lichtman et al. 2003). This should be in the form of workshops focusing on teaching and assessment practices, preparation of teaching material and assessment of students (Luhanga 2018:135; RNAO 2016:26).

*Planning for placement of nursing students to the clinical sites* is vital as this is where undergraduate nursing students acquire the knowledge and skills that enable them to be competent nurses (RNAO 2016:26). The role of the nurse educator is to assess suitability of clinical placements, as determined by availability of learning opportunities. This is in response to the fact that provision of high-quality clinical supervision is the responsibility of the nurse educator (RNAO 2016:25).

### Theme Two: Facilitation of nursing students’ clinical placements

In order for facilitation of nursing students’ clinical placements to be effective, as identified in 34 papers (Adibelli & Boyaci 2018; Akram, Mohamad & Akram 2018; Ajani & Moez 2011; Barrett 2007; Bentley & Pegram 2003; Brown et al. 2005; Butler et al. 2011; Cangelosi, Crocker & Sorrell 2009; Carlson, Wann-Hansson & Pilhammar 2009; Carlson, Pilhammar & Wann-Hansson 2010; Duffy 2009; Ehrenberg & Haggblom 2007; Farzi, Shahriari & Farzi 2018; Foley, Myrick & Yonge 2012; Frazer et al. 2014; Henderson et al. 2006; Hendricks et al. 2013; Hossein et al. 2010; Huybrecht et al. 2011; Kpodo 2015; Lambert & Glacken 2005; Lee, Cholowski & Williams 2002; Leonard, McCutcheon & Rogers 2016; Luhanga et al. 2010; Luhanga, Yonge & Myrick 2008; Matthew-Maich et al. 2015; McSharry et al. 2010; Msiska, Munkhondya & Chilemba 2014; Öhrling & Hallberg 2001; Paton 2005, 2007; Price et al. 2011; Raisler, O’Grady & Lori 2003; RNAO 2016; Udlis 2008), nurse educators should incorporate orientation of nursing students to clinical practice, planning for clinical teaching and the clinical teaching process, respectively.

*Orientation of nursing students to clinical practice* is vital in ensuring that they become informed about the nature of clinical practice. The orientation meeting between the nurse educator and nursing students at the beginning of the teaching programme, before clinical placement, should inform the students about the clinical practice requirements, practical skills to be practised and assessment methods (Farzi et al. 2018). This meeting should inform the student about what to expect at a particular placement and the clinical activities that are within their scope of practice and level of education (RNAO 2016:28).

*Planning for clinical teaching* implies that a well-coordinated programme of clinical placements should be prepared that takes into consideration correlation of theory to practice. This programme should encompass pre-contact preparation of the necessary documents – for example, copies of programme learning objectives, clinical assessment forms and feedback tools to be used in clinical teaching of nursing students (Kpodo 2015:79; Matthew-Maich et al. 2015:45). Of importance is recognition of previous experiences and learning needs of nursing students, finding out about their learning capabilities and making available appropriate learning resources (Hossein et al. 2010:8; RNAO 2016:28), which could include technology that facilitates clinical teaching and learning (Adibelli & Boyaci 2018:734).

Three aspects of the *clinical teaching process* were identified in this review – namely, maintenance of clinical credibility; bridging the theory-practice gap and use of an appropriate clinical teaching model.

Firstly, nurse educators should maintain their clinical credibility by being clinically current, and by making time to learn techniques for sharing knowledge, coaching and supporting others in their learning. Clinical currency, as evidenced by the recency of clinical experience is viewed as being clinical credibility (McSharry et al. 2010:190; Msiska et al. 2014:844). Furthermore, a clinically credible nurse educator ensures that the knowledge received by nursing students is applicable to clinical practice (Leonard et al. 2016:15).

Secondly, nurse educators involved in clinical teaching of nursing students should assist in bridging the theory-practice gap by providing clinical education to nursing students that enhances their application of theory in the conduct of their clinical practice, thus building the knowledge, skills and attitudes essential for professional practice (Ajani & Moez 2011:3927; Akram et al. 2018:876; Barrett 2007:367; Bentley & Pegram 2003; Ehrenberg & Haggblom 2007:67; Leonard et al. 2016:2). However, the theory-practice gap is viewed as posing a challenge in the light of the multifaceted role of nurse educators and multiplicity of clinical teaching styles used in clinical teaching (Matthew-Maich et al. 2015:50).

The included papers highlight four clinical teaching models which can be used in clinical teaching. The first, the traditional facilitation model aimed to utilise a nurse educator in clinical teaching in view of both academic and clinical expertise (dual role). However, the sharing of responsibilities by these nurse educators, coupled with a heavy workload, limited the effectiveness of the clinical teaching role (Leonard et al. 2016:5). The second clinical teaching model, the preceptorship model, requires that the student is assigned to a registered nurse on a one-to-one basis. This is aimed at developing the professional knowledge and skills of nursing students in clinical practice, preparing them for their role-transitioning from student to graduate nurses through role modelling and feedback (Brown et al. 2005:84; Butler et al. 2011:298; Carlson et al. 2009, 2010; Duffy 2009:166; Foley et al. 2012:1; Frazer et al. 2014; Henderson et al. 2006; Price et al. 2011:780; Udlis 2008:20). The third model, the mentorship model, involves a qualified nurse being used to supervise nursing students on a one-to one basis to teach nursing students to expand their (practical) skills, overcome obstacles and build on their strengths to make positive choices and develop their practical skill so that they could become knowledgeable and well-rounded professionals. However, the workloads of and support required by mentor nurses should be carefully considered so that they may perform their mentoring function competently (Cangelosi et al. 2009:367; Huybrecht et al. 2011:274; Leonard et al. 2016:4; Luhanga, et al. 2008:227, 2010:1; Öhrling & Hallberg 2001:530; Paton 2005, 2007; Raisler et al. 2003:398). The fourth clinical teaching model evident in the literature is that of an established dedicated education unit seeking to provide a positive clinical education environment for nursing students, facilitators and educational staff (Lambert & Glacken 2005:664; Lee et al. 2002:412).

The nurse educator to student ratio is viewed as vital in clinical teaching of nursing students. These ratios, as they relate to the clinical supervision models, are, for example, as follows: preceptor and mentorship model, 1:1, whilst the facilitation model has a ratio of 1:6 to 1:8 (Hendricks et al. 2013).

### Theme Three: Evaluation of students’ clinical skills

Evaluation of students’ clinical skills, mentioned by five papers, encompasses reflection by the nursing student on the clinical learning experiences, and feedback given by the nurse educator on the level of clinical competence of the nursing student.

Undergraduate nursing students are expected to *reflect* on practice events and skills that they encounter during their learning process in clinical settings. By reflecting on their own learning, students begin to identify the strengths and weaknesses in their work. Reflection has been found to facilitate development of autonomy, open mindedness, critical thinking and sensitivity in nursing students (Da Silva & Almeida de Figueiredo 2017:4118; Frazer et al. 2014:4; Scully 2011:93).

*Feedback* is a two-way process in which the nurse educator shares with the student information based on observation, with the aim of enabling the student to reach the defined goal and of informing the student about areas of improvement in clinical practice (McCarthy & Murphy 2008:301). Feedback needs to be constructive, unbiased and timely, to provide direction that would increase motivation, confidence, self-esteem, cognitive skills and behaviours and to make reference to course learning outcomes (McCarthy & Murphy 2008:301). It is important for nurse educators to model the behaviour they wish to see in students when giving feedback (McCarthy & Murphy 2008:301; Phillips et al. 2017:205).

### Theme Four: Modelling professional clinical practice

The aspects covered under the theme of *modelling professional clinical practice,* as identified in 12 papers, are: emotional intelligence, self-evaluation, role modelling and continuous professional development.

*Emotional intelligence* is the ability of nurse educators to control their own emotions whilst influencing the other person – for example, the student – to act in an acceptable manner (Allen, Ploeg & Kaasalainen 2012:231). Two complementary models of emotional intelligence are in existence, namely, trait and ability models, both of which enable nurse educators to demonstrate their own level of maturity. The trait model enables the nurse educator to contain self in a stressful clinical environment, whereas the ability model is used to assess and solve emotional problems on the part of nursing students (Allen et al. 2012:231).

*Self-evaluation* covers self-reflection ((in)formal self-assessment) by nurse educators of knowledge, skills and performance (Lichtman et al. 2003:455; Little & Milliken 2007; Parkinson 2016:9; Phillips & Vintern 2010:226; Schub & Heering 2016:3). *Peer evaluation* entails a formal or informal evaluation of the performance of the nurse educator conducted by a peer of equal status or a senior, such as, for example, head of department (Landers 2015:13; Parkinson 2016:9; Schub & Heering 2016:2). *Student evaluation* refers to reliable feedback about the quality of clinical teaching students received at various levels of their education programme. This requires creativity on the part of nurse educators in obtaining feedback from nursing students (Parkinson 2016:9) with a view to improving the practice of nursing students.

*Role modelling* professional behaviour is essential in clinical teaching to help professional development of students. Students evaluate role modelling of clinical teachers according to the quality of clinical teaching they receive from them, and their attitude towards the students. It is crucial for nurse educators to understand the characteristics of their students so as to be able to adapt their teaching strategies accordingly (Canadian Nurses Association 2005; Hart 2017:256).

*Continuous professional development:* an inherent part of the nurse educator role is to be up-to-date with nursing developments as these are a crucial component of clinical teaching (Leonard et al. 2016:149). Effectively structured professional development for nurse educators includes providing update sessions, resource and education packages and interactive workshops (Esmaeili et al. 2014; RNAO 2016:27).

### Theme Five: Work-based assessment in the clinical environment

Work-based assessment of competence of nursing students is crucial in maintaining professional standards, as identified in six papers. This includes the assessment process as well as clinical assessment tools. Areas of improvement in the performance of nursing students are identified during this process (Schub & Heering 2016:2).

Problems identified by Rafiee et al. (2014:44) with regard to the *assessment process* include lack of assessment instruments to do formative assessment, resulting in inability to conduct appropriate formative assessment; clinical assessments not performed in a timely manner by nurse educators and assessment practices not standardised both at national and international level. Helminen et al. (2016) reported that assessment practices vary at different nursing education institutions. Rafiee et al. (2014:45) assert that there is a need to upgrade the current clinical assessment forms, and nurse educators should improve their knowledge about what is entailed in a complete and comprehensive clinical assessment.

Helminen et al. (2016:309) state that the clinical assessment process entails formative assessment (relating to ongoing process, which lasts throughout clinical education) and summative assessment (which can be used both at the end of every nursing student’s clinical practice period and at the end of the programme of studies, before graduating).

Various authors refer to the *clinical assessment tools* that have been used in nursing over the years. These include: the portfolio (a collection of evidence by the individual being assessed to demonstrate acquisition and maintenance of skills, knowledge and attitudes); direct observation (a visual assessment conducted by the nurse educator as the student performs specific tasks, using a checklist); Objective Structured Clinical Examination (participation in a series of structured activities that test knowledge and skill in a variety of clinical areas, allowing participants to practise skills in a controlled setting); interviews (an interpersonal process that enables students to demonstrate their ability to integrate their knowledge, skills and attitudes to preceptors); reflective journal (which is student-centred and promotes the students’ critical thinking and analytical abilities, thus contributing to their development as professionals) and rating scale (a valid and reliable tool that is most useful for summative evaluation of student performance) (Kpodo 2015:80; Marchigiano, Eduljee & Harvey 2011:143; Nulty et al. 2011:145; Schub & Heering 2016:4;).

### Theme Six: Clinical teaching in the simulation laboratory

Clinical teaching in the simulation laboratory was supported by eight papers. Simulation is a technique that can be used to replace real experiences with guided experiences that replicate substantial aspects of the real world in a fully interactive manner. Simulation has been used primarily in undergraduate nursing programmes to teach nursing skills in view of its safety and ability to afford nursing students the opportunity to practise skills until they achieve competency level. Simulation complements traditional education with actual patients and enables nursing students to learn in ways that eliminate risks to patients, thus providing them with opportunities to develop and explore problem-solving skills, clinical skills and critical thinking skills (Berragan 2014:1143; Cant & Cooper 2010:3; Khalaila 2014:253; McCaughey & Trayner 2010:827; Mills et al. 2014:12; Ribeiro et al. 2018:451; Sanford 2010:1006; Secomb, McKenna & Smith 2012:3475).

## Discussion

The six themes that emerged from this integrative review reflected that clinical teaching is a process, including various aspects.

By examining the first theme – *planning for clinical teaching* practice – it was found that the nurse educator should prioritise self-preparation and planning for clinical placement. Self-preparation is about identifying own knowledge gaps and engaging in in-service education on a continuous basis. The areas that should be strengthened are clinical teaching and assessment methods, leadership and reflective thinking (Duffy & Watson 2001:551; Kpodo 2015:79). Planning also includes the assessment that has to be made by nurse educators with regard to the suitability of clinical sites. This should enable nurse educators to monitor the downgrading and upgrading of clinical sites that is often done by health authorities, especially in view of the fact that lack of suitable clinical placement sites leads to competition for clinical placement areas, and to ensure that students receive clinical learning experiences that are aligned to their theoretical learning (Muthathi, Thurling & Armstrong 2017:6).

The second theme – *facilitation of nursing students’ clinical placements –* focused on implementation of the clinical teaching process. This theme was supported by the majority of papers reviewed. A well-structured clinical teaching programme was found to be necessary. A well-structured clinical programme would be evident in the resources provided to nursing students to utilise during their clinical exposure (Luhanga 2018:132). Furthermore, the clinical sites should be given the necessary communication that informs them about the clinical learning experiences and clinical hours required by nursing students (Kpodo 2015:78; Taniyama, Kai & Takahashi 2012).

The situation in the clinical sites should be monitored on a continuous basis in order to enhance the quality of clinical education (Farzi et al. 2018), given that nursing students should be placed at clinical sites where their learning needs will be met (Muthathi et al. 2017:2). Facilitation of nursing students’ clinical placements also includes establishment of clinical credibility and ensuring bridging of the theory-practice gap (Dadgaran, Parvizy & Peyrovi 2012; Muthathi et al. 2017:7; Shoghi et al. 2019:1).

Nurse educators must play a supportive role and be actively involved in clinical teaching in order to be familiar with what is happening in the clinical setting. They should be able to implement appropriate clinical teaching models in order for students to receive clinical teaching that is relevant and aligned with their learning objectives and level of teaching received (Leonard et al. 2016:150; Meskell, Murphy & Shaw 2009:784; Muthathi et al. 2017:6).

The traditional facilitation model as one of the four clinical teaching models highlighted in the included papers is often regarded as the ‘gold’ standard (Luhanga 2018:125). However, for this model to be effectively implemented, increasing the number of nurse educators with relevant education in clinical practice and expertise may be required (Bvumbwe & Mtshali 2018:9).

The third theme – *evaluation of students’ clinical assessment skills –* related to the interaction between the nurse educator and nursing student, with the focus on reflection by the student and feedback by the nurse educator. This was confirmed by Karimi et al. (2017:5195) who found that reflection on, for example, clinical skills enables nursing students to share their own strengths and weaknesses with the nurse educator, thus increasing the quality of care provided by nursing students to patients. With regard to feedback, the absence of feedback confirms lack of support and clinical supervision in clinical practice. Feedback should be timely, constructive and done in a respectful manner (Kamphinda &Chilemba 2019:7; Kok & Chabeli 2002:35; Montes, Rodrigues & Azevedo 2019:667).

The fourth theme – *modelling professional clinical practice –* highlights the four characteristics of professional clinical practice that are interconnected. These are emotional intelligence, self-evaluation, role modelling and continuous professional development. Emotional intelligence enables the clinical teacher to pursue self-evaluation (Muthathi et al. 2017:7).

The process of role modelling attracts mutual interaction of clinical educators and nursing students. It enhances humanistic and professional growth. Humanistic growth is ensured through intellectual, spiritual and emotional development. A competent clinical teacher utilises role modelling to council, guide and promote students’ competency (Nouri et al. 2014). Role modelling and continuous professional development go hand in hand, as continuous professional development needs to happen in an ever-changing clinical environment for the nurse educator to demonstrate (role-modelling) the correct practices to the students (Muthathi et al. 2017:7).

The fifth theme – *work-based assessment in the clinical environment –* highlights the importance of clinical assessment in development of nursing students as competent professional nurses. Work-based assessment is an integral part of clinical teaching. It provides the students with an opportunity to develop critical thinking and problem-solving skills whilst taking care of patients. Hence, it is referred to as an authentic assessment (Almalkawi 2019). The extent of readiness of nursing students for any type of assessment should be promoted, and appropriate tools should be used for each assessment (Almalkawi 2019:246; EdCan National Education Framework Cancer Nursing 2008).

The sixth theme – c*linical teaching in the simulation laboratory –* highlights the fact that simulation-based learning is vital for undergraduate nursing education, particularly in a context fuelled by the shortage of clinical faculty and diminishing the number of clinical sites (Aebersold 2018). Simulation-based learning provides undergraduate nursing students with an opportunity to practise responding to rare emergency situations and authentic life situations in a safe environment. It affords the students a level of competence through the immediate feedback they receive through debriefing and the opportunity for repetitive practice (Aebersold 2018; Dreifuerst 2009:109; Kim, Park & Shin 2016). A nurse educator should be trained to use simulation (such as computerised low/medium/high fidelity mannequins, role-play, standardised or simulated patients and virtual simulations) in clinical teaching (Powell, Scrooby & Van Graan 2020).

The weakness of an integrative literature review is that both research, such as randomised controlled trials, quantitative, qualitative, mixed methods as well as non-research papers, such as editorials and opinion papers, are included. Non-research papers are often regarded a lower level of evidence according to various hierarchies of evidence (Murad et al. 2016). This review found a variety of levels of evidence on the topic. However, more high-level evidence studies (such as randomised controlled trials) need to be done on the clinical teaching practices of nurse educators as only one randomised controlled trial was identified. Furthermore, there is a need for studies to be conducted in resource-constrained settings in developing countries as the majority of papers were from developed countries, such as Canada, Australia and the United States of America.

## Conclusion

This review aimed to summarise the best clinical teaching practices of nurse educators, teaching undergraduate nursing programmes. The evidence obtained from this review points to the existence of various practices with regard to clinical teaching. More clinical trials need to be conducted on clinical teaching practices and in resource-constrained settings.
